# Key steps to reduce the aerosolized risk related to total laryngectomy in COVID‐19 era: A case report

**DOI:** 10.1002/ccr3.3601

**Published:** 2021-06-19

**Authors:** Robert Chessman, Tahwinder Singh, Davinia Passley, Richard Gande, Katarzyna Konieczny, Nimesh N. Patel

**Affiliations:** ^1^ ENT Surgery University Hospital Southampton NHS Foundation Trust Southampton UK

**Keywords:** aerosolization, COVID‐19, laryngectomy, larynx

## Abstract

Laryngectomy surgery is a highly aerosolizing procedure, and we document the key steps, including the addition of a novel Perspex shield, which can be enacted during the COVID‐19 pandemic to reduce risk to the patient and healthcare professional.

## Graphical abstract

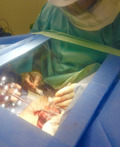

## 1 INTRODUCTION

Total laryngectomy is an aerosol‐generating procedure. End tracheostomy bypasses important airway protective anatomical structures. Laryngectomees are regarded as high risk for life‐threatening coronavirus infection.^1^ Laryngectomy therefore poses significant risks to the patient and healthcare workers when performed during a respiratory virus pandemic. Nevertheless, is it often a procedure that cannot be deferred.

There is evidence about the benefits of testing, isolation, surgical preparation, and personal protective equipment; we thus applied these principles in planning and conducting the procedure. However, due to the unique nature of the surgical procedure, the highest risks of aerosolization exist intraoperatively during the formation of the tracheotomy and when creating the end tracheostome. We therefore focused on modifying this part of the procedure to minimize the duration of an open airway and mitigate the risk to the healthcare professionals. There is only one other published article relating to a modified technique for performing a laryngectomy during the COVID‐19 pandemic.^2^ This case report offers a different approach which is easily reproducible, and we believe adds further protection to the healthcare workers involved during total laryngectomy.

## 2 CASE SUMMARY AND DISCUSSION

A 70‐year‐old man presented with upper airway obstruction and aspiration pneumonia due to a lesion fixing his larynx and invading his subglottis. His airway was stabilized by endoscopic tumor debulking and representative biopsies were taken. These demonstrated moderately differentiated squamous cell carcinoma. Radiological staging confirmed the lesion as T4N1M0 laryngeal (glottic) cancer by the Union for International Cancer Control (UICC staging. Following full discussion between the patient and the local Head and Neck Multidisciplinary Team (MDT), proceeding during the COVID‐19 pandemic, total laryngectomy and bilateral neck dissection were felt to be the most appropriate treatment option. Nonsurgical and palliative treatments were discussed in detail prior to full informed consent being taken for surgery. There was a specific discussion regarding the risks of major surgery during the coronavirus pandemic.

As per BAHNO (British Association of Head & Neck Oncologists) guidelines,^1^ two COVID‐19 antigen nasopharyngeal swabs were taken at interval in the 2 days prior to surgery and were negative. The patient was advised to isolate for 1 week prior to surgery.

Due to the high‐risk nature of the procedure and the current concern over accuracy of the available tests, the patient was managed throughout the peri‐operative period on the assumption that they may have been COVID‐19 positive. All appropriate precautions were therefore adopted in the peri‐operative period.

Prior to surgery, a full theater team brief took place, as a part of the World Health Organization (WHO) checklist, to ensure all staff were aware that the surgical procedure was aerosol‐generating and the new additional steps involved for the procedure to try to minimize risk to the team were discussed.

The entire theater team involved in the case donned full personal protective equipment (PPE) and FFP3 respirator masks prior to entering the theater, as recommended in Public Health England's guidelines on aerosolizing procedures.^3^ The anesthetic team followed their well‐rehearsed COVID‐19 intubation procedure in the operating room.

Initially, a dilute 50:50 wash with povidone‐iodine disinfectant and saline was used in the oral cavity to destroy potential virus in the upper aerodigestive tract prior to performing panendoscopy to assess the tumor.^4^, ^5^

With an aim to minimize aerosolization of secretions, bilateral neck dissections and mobilization of the larynx was performed first without any breach of the airway. The stoma site was then prepared, and vicryl stomal sutures were placed in the skin ready to attach to the trachea.

**FIGURE 1:**
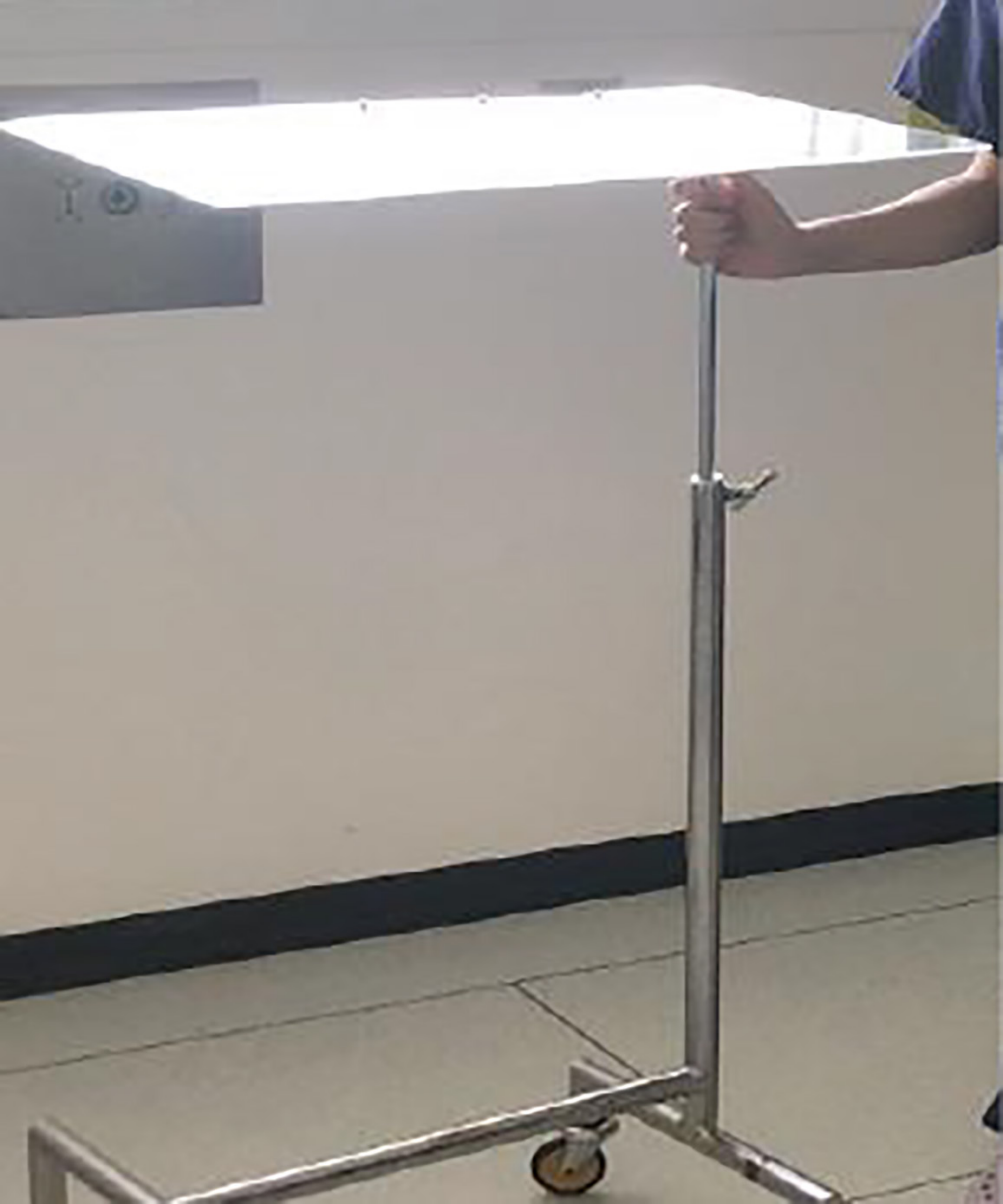
Perspex screen mounted to the Mayo table base

**FIGURE 2:**
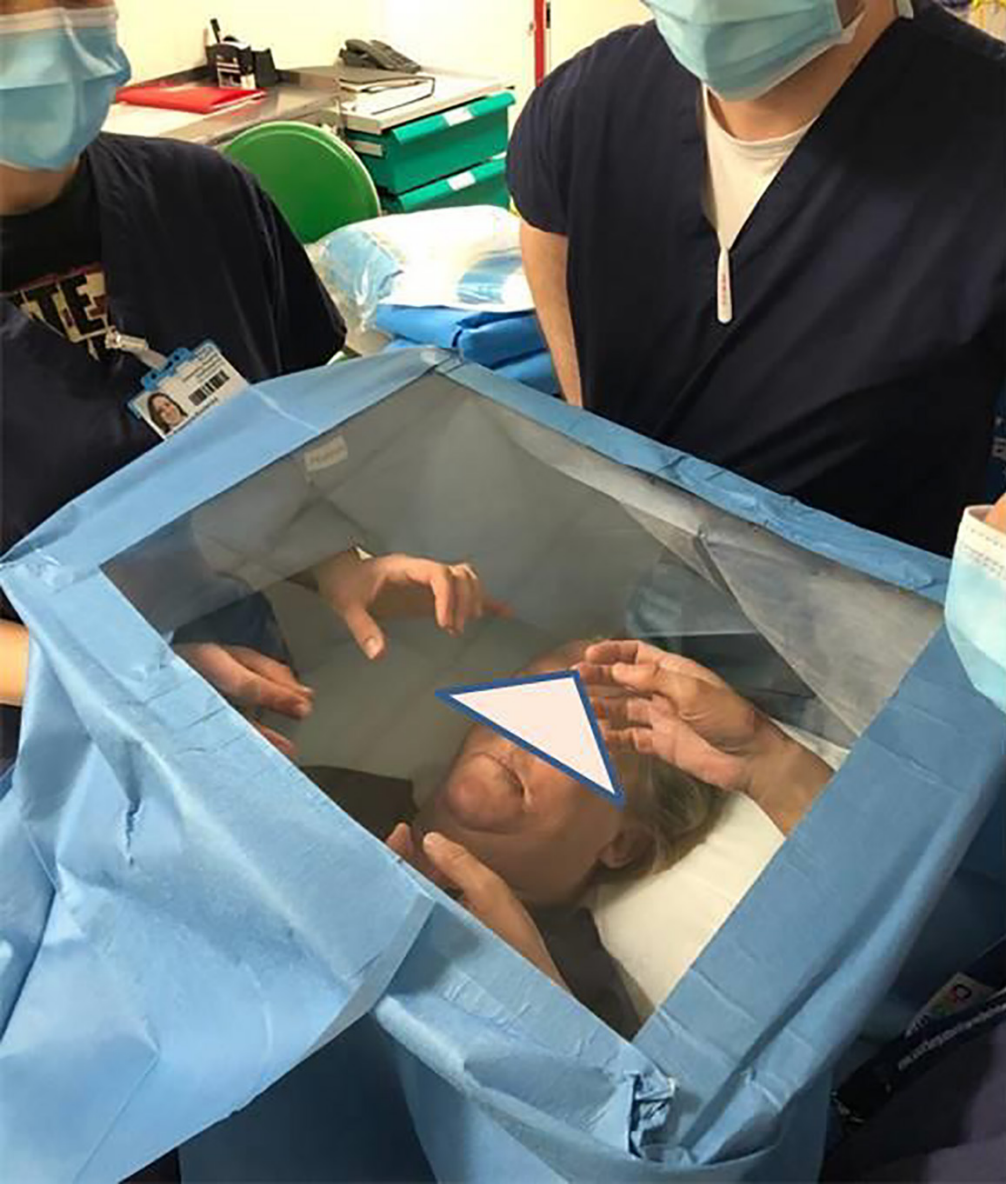
Demonstration of draping of cheap locally manufactured Perspex operative shield

**FIGURE 3:**
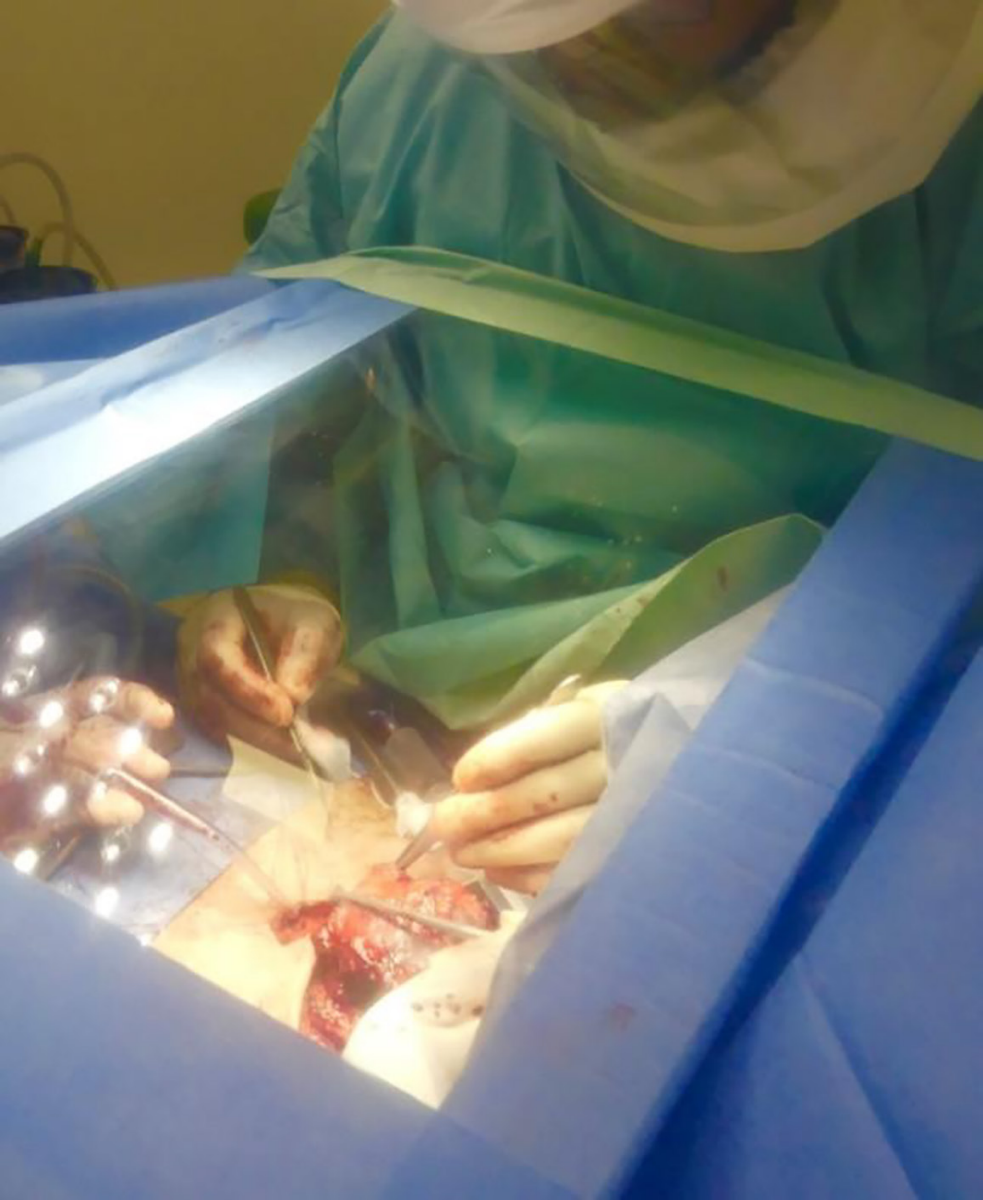
Perspex modified Mayo table for tracheostomy

During the course of pre‐operative planning, an inexpensive Perspex shield which could be used intraoperatively to reduce fomite and micro‐droplet spread during high‐risk parts of the procedure was commissioned with local engineers.^6^ The shield was designed to allow free movements of hands with surgical instruments while allowing clear visualization of the operative site (Figures 1 and 2). Once the surgical site was fully prepared the theater team, and anesthetist were notified of imminent airway changeover. The purpose‐made clear Perspex shield, attached to a Mayo table base, was moved into position over the patient. Sterile surgical side drapes were attached for further protection from aerosolized contamination (Figure 3). The Perspex shield could be swung away from the patient as necessary or in the case of an emergency.

**FIGURE 4:**
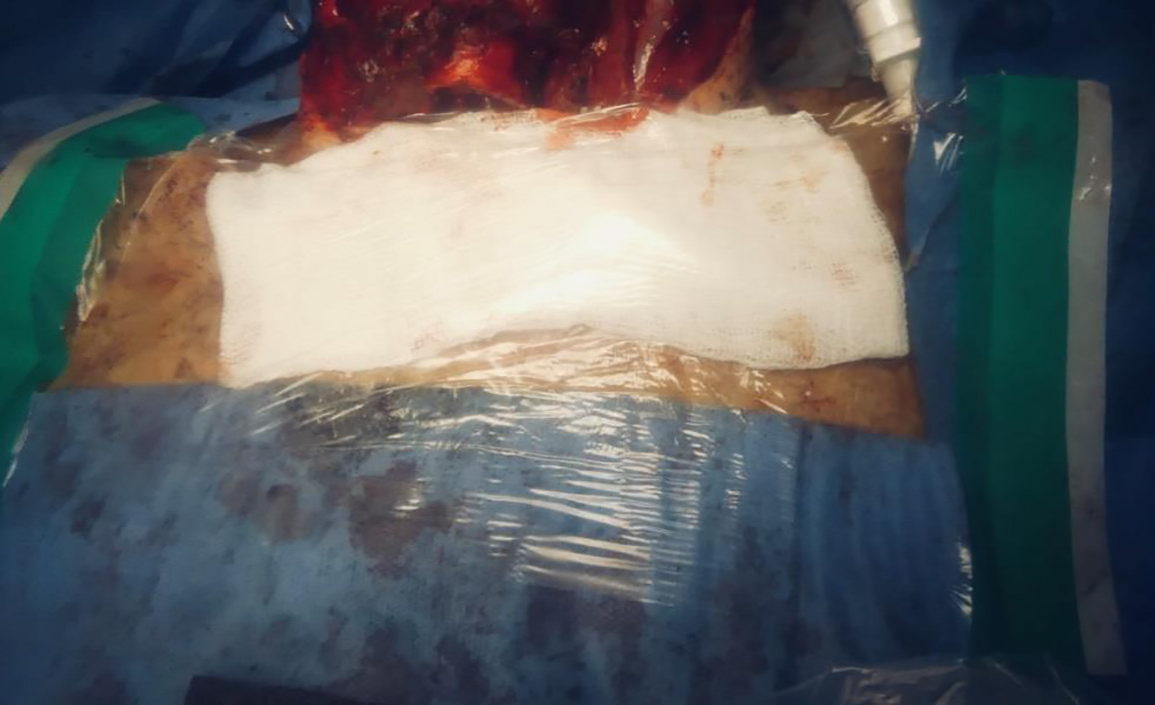
Closed system after tracheostomy with Rusch endotracheal tube in place

Pre‐oxygenation was performed with 100% oxygen for 3 minutes, and the endotracheal tube advanced to avoid inadvertent cuff incision and so as to maintain a closed airway system for as long as possible. Positive‐pressure ventilation and gas delivery were stopped, and continuous suction of the airway with a wide‐bore sucker was performed during incision of the trachea to minimize aerosolization. Operating through a screen with limited space was unfamiliar and challenging. Therefore, to minimize risk to the patient and surgeons, care had to be taken to ensure safe surgical technique throughout. A J‐endotracheal tube (Rusch) was inserted to the stoma site, cuff inflated, and ventilation was resumed, while carefully observing for any airway leak. The trachea was secured to the skin with the preprepared sutures. Once the end tracheostome was completed, a large Tegaderm transparent film dressing was placed over a moist gauze to seal off the stoma (Figure 4). Only once the team felt the airway was secured, and sealed was the Perspex shield mounted onto the Mayo table base removed from the operating field. The larynx was then excised and the neopharynx could be formed. Despite potentially infected mucosa still being exposed at this point, as the patient was now ventilating on a closed circuit again, the risk of aerosolization was lower, and therefore, it was felt the Perspex shield was not needed. Staple closure of the pharynx was performed as part of our standard laryngectomy procedure. De Seta et al describe a modification to this technique to ensure minimal exposure to the aerodigestive tract which could be adopted in the future.^2^ Primary trachea‐esophageal puncture was not performed. It was noted on inspection following the procedure that significant aerosolization of blood and secretions were evident on the under surface of Perspex table.

At extubation, following discussion with the anesthetic team, a well‐fitting adhesive base plate and Heat and Moisture Exchanger (HME) was placed over the new stoma site, in order to minimize environmental contamination with airway secretions. The airway was thoroughly suctioned prior to transfer to a recovery area separated from general recovery.

The patient was to be nursed in a side room on a ward for non‐COVID‐19 patients. PPE was used during all nursing and medical care for the patient. The patient was discharged home with full discharge planning and community support.

## 3 CONCLUSION

This case report aims to document a technique which can be used during the COVID‐19 era when performing a laryngectomy to reduce risk to the patient and healthcare professional. The steps described in Table 1 are simple to learn and easily reproducible. The main limitation of this procedure was the restrictive nature of operating under the Perspex table. However, with practice, this step becomes more effective and we believe gives an important additional level of protection during the evolving COVID‐19 pandemic.

**Table 1:** Key steps when performing a laryngectomy in the COVID‐19‐era

1. Pre‐operative isolation and testing
2. Specific team briefing as part of Peri‐operative WHO Checklist highlighting the risks involved
3. Full PPE for all theater team
4. Dilute 50:50 wash with Povidone‐iodine disinfectant and saline in the oral cavity
5. Perform neck dissection and mobilization of the larynx prior to breaching airway
6. Prepare stoma site with sutures in place
7. Cover operative field with Perspex shield
8. Pre‐oxygenation of patient
9. Cessation of Positive‐pressure ventilation and gas delivery
10. Perform tracheotomy and secure sutures
11. Insert Rusch tube, ensure ventilation and cover with large Tegaderm dressing
12. Complete formation of neopharynx
13. On extubation fit base plate and HME over the new stoma site

## CONFLICT OF INTEREST

None declared.

## AUTHOR CONTRIBUTIONS

RC: leads in writing the manuscript. TS: helped to develop the technique described, provided critical feedback and revised the manuscript. DP: helped to develop the technique described, provided critical feedback, and revised the manuscript. RG: helped to develop the technique described, provided critical feedback, and revised the manuscript. KMK: helped to develop the technique described, provided critical feedback, and revised the manuscript.

## ETHICAL APPROVAL

No ethical approval required.

## References

[1] Kerawala C . Proposed guidance for laryngectomy surgery during the COVID‐19 pandemic. British Association of Head & Neck Oncologists. https://www.bahno.org.uk/bahno_laryngectomy_guideance_during_covid‐19_pandemic.aspx. Published March 24, 2020. Accessed March 31, 2020

[2] De Sata D , Marrosu V , Russo F , Carta F , Puxeddu R . Closed total laryngectomy during the COVID‐19 pandemic disease. Laryngoscope. 2020;130(11).2622–2624. 32570286 10.1002/lary.28919 PMC7361463

[3] Public Health England . COVID‐19 Personal protective equipment (PPE). GOV.UK. https://www.gov.uk/government/publications/wuhan‐novel‐coronavirus‐infection‐prevention‐and‐control/covid‐19‐personal‐protective‐equipment‐ppe. Published April 24, 2020, Updated May 3, 2020. Accessed May 4 2020

[4] Eggers M , Koburger‐Janssen T , Eickmann M , Zorn J . In vitro bactericidal and virucidal efficacy of povidone‐iodine gargle/mouthwash against respiratory and oral tract pathogens. Infect Dis Ther. 2018;7(2):249‐259. 29633177 10.1007/s40121-018-0200-7 PMC5986684

[5] Kirk‐Bayley J , Combes J , Sunkaraneni S , Challacombe S . The use of povidone iodine nasal spray and mouthwash during the current COVID‐19 pandemic may reduce cross infection and protect healthcare workers. SSRN: https://ssrn.com/abstract=3563092 or 10.2139/ssrn.3563092. Published March 30, 2020. Updated April 24, 2020. Accessed April 30, 2020

[6] Konieczny K . Tracheostomy in the COVID era‐ A Customised PERSPEX table to reduce the risk of aerosolization. ENT UK. https://www.entuk.org/tracheostomy‐covid‐era‐customised‐perspex‐table‐reduce‐risk‐aerosolisation. Published May 7, 2020

